# Therapeutic advances in obesity management: an overview of the therapeutic interventions

**DOI:** 10.3389/fendo.2024.1364503

**Published:** 2024-04-23

**Authors:** Moody Al Roomy, Kainat Hussain, Hawraa M. Behbehani, Jenna Abu-Farha, Rayan Al-Harris, Aishwarya Mariam Ambi, Mohammed Altigani Abdalla, Fahd Al-Mulla, Mohamed Abu-Farha, Jehad Abubaker

**Affiliations:** ^1^ Department of Biochemistry and Molecular Biology, Dasman Diabetes Institute, Kuwait City, Kuwait; ^2^ Department of Translational Research, Dasman Diabetes Institute, Kuwait City, Kuwait; ^3^ Hull York Medical School, University of Hull, Hull, United Kingdom

**Keywords:** obesity, GLP-1 RAs, tirzepatide, bariatric, weight loss

## Abstract

Obesity has become a global epidemic in the modern world, significantly impacting the global healthcare economy. Lifestyle interventions remain the primary approach to managing obesity, with medical therapy considered a secondary option, often used in conjunction with lifestyle modifications. In recent years, there has been a proliferation of newer therapeutic agents, revolutionizing the treatment landscape for obesity. Notably, glucagon-like peptide-1 receptor agonists (GLP-1 RAs), such as semaglutide, liraglutide, and the recently approved dual GLP-1/GIP RAs agonist tirzepatide, have emerged as effective medications for managing obesity, resulting in significant weight loss. These agents not only promote weight reduction but also improve metabolic parameters, including lipid profiles, glucose levels, and central adiposity. On the other hand, bariatric surgery has demonstrated superior efficacy in achieving weight reduction and addressing overall metabolic imbalances. However, with ongoing technological advancements, there is an ongoing debate regarding whether personalized medicine, targeting specific components, will shape the future of developing novel therapeutic agents for obesity management.

## Introduction

Obesity is defined as excessive or abnormal accumulation of body fat both centrally and subcutaneously and presents risks to health. Over the past decades, the prevalence of obesity has significantly increased at an alarming rate putting a strain on the world’s economy (1). Traditionally, obesity was defined as an increase in body weight by at least 20% of the ideal body weight. In today’s world, obesity is classified merely based on measuring the body mass index (BMI), calculated as body weight in kilograms (kgs) divided by the squared meter (m^2^) of the height. Thus, normal body weight is when the BMI ranges between 18.5-24.9 kg/m^2^, whereas a BMI ranging from 25-29.9 kg/m^2^ is considered overweight while ≥ 30 kg/m^2^ is obese (2). Therefore, based on the aforementioned parameters, the World Health Organization (WHO) reported that in 2017 nearly two billion adults aged ≥ 18 years were overweight of whom over 600 million were obese and it was declared as a health crisis of the 20^th^ century (3). The rate is high among adults at around 27.5% and 47.1% in children (4). Geographically, America and Europe have the highest rates of obesity as the rates increased from 6.8% in 1980 to 22.4% in 2019 in America. In addition, the prevalence of obesity in Europe increased from 8.4% in 1980 to 20% in 2019 (5).

**Figure 1:**
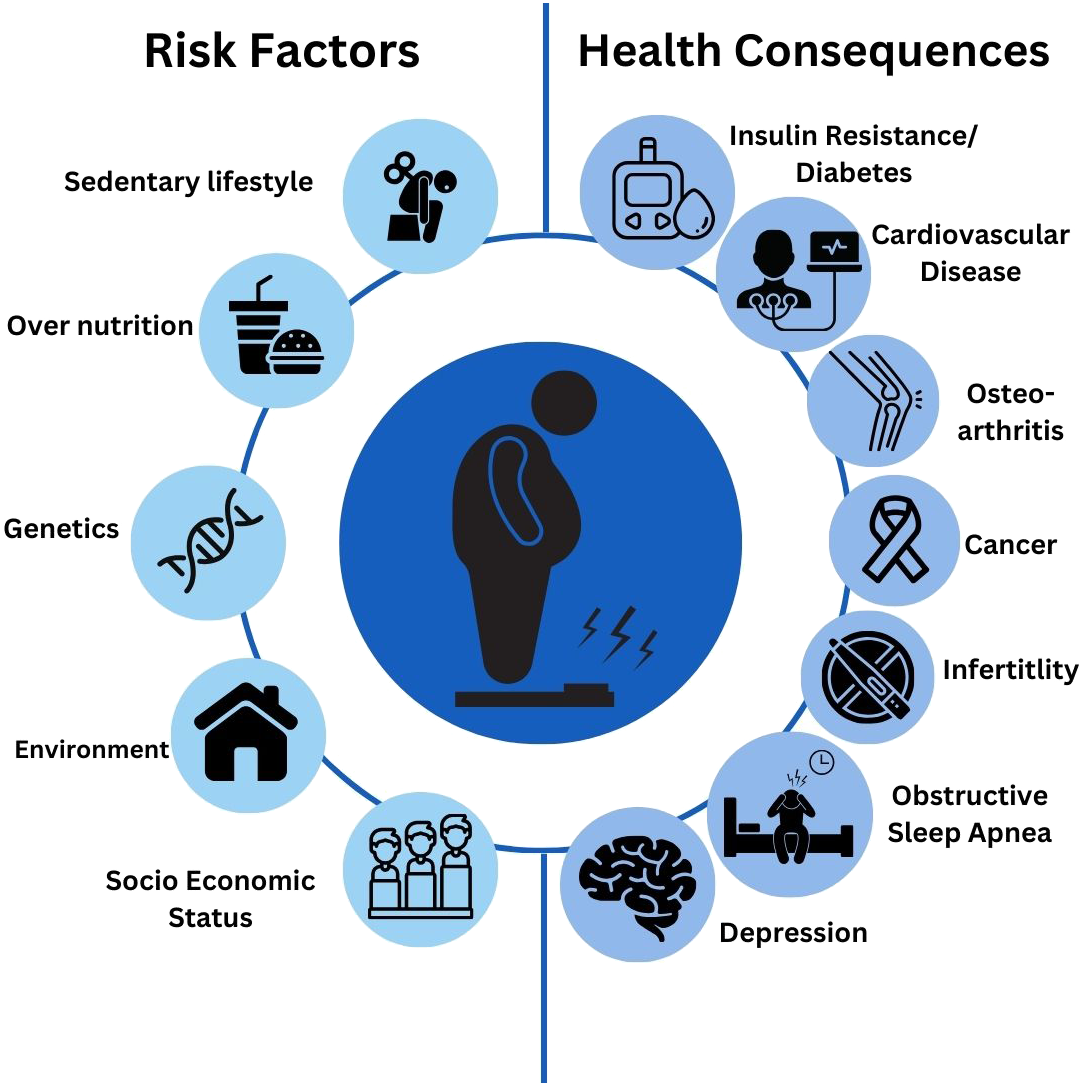
Risk factors and health consequences associated with obesity.

Obesity is a multi-factorial disease caused by a complex of genetic, environmental, and behavioral factors (**Figure 1**). Interaction between these factors contributes to the complexity of obesity and makes its treatment more challenging as the result of the complex interaction among different genes and other risk factors such as environmental and lifestyle factors (6). Although an individual’s genetic background is one of the essential factors determined as a cause of obesity, the basis of obesity is not genetic (7). The evidence suggests that genes often need to be closely linked with environmental and lifestyle risk factors to affect weight (8). Therefore, further understanding the common causes of obesity and weight gain is crucial. Recently, several genetic factors contribute to the predisposition of obesity, and several genes that regulate body weight and metabolism have been identified. For example, the fat mass and obesity (FTO) associated gene and the melanocortin 4 receptor (MC4R) gene have been associated with increased body weight and cause obesity; however, it is important to note that genetics alone cannot determine obesity (9, 10). On the other hand, at the global scale, obesity is caused by media devices (smartphones, video games, computer monitors, and television sets) and a sedentary lifestyle which in turn aided by certain unhealthy dietary patterns such as increased portion size, sugar beverages, junk food, and low activity level (11). Children and young adults with constant exposure to media devices are typically inactive and may use less energy which will disrupt their appetite signaling and cause them to eat even more than is needed (12). Moreover, obesity increases the risk of chronic diseases, such as diabetes mellitus and cardiovascular disease. It also reduces the overall quality of life and increases the risk of cancer with a detrimental impact on individuals and societies. According to data from the global burden of disease, in 2017 there were around four million deaths globally all attributed to diabetes and cardiovascular disorders (13). Therefore, it is important to find an effective treatment for people who are overweight or obese and it is also essential to implement preventive measures and policies for those who are having normal BMI (**Table 1**). Furthermore, to reduce the prevalence of obesity and to lessen the burden of obesity-related chronic diseases, effective preventative interventions are required to make the fight against obesity a top priority which can improve population health, lower healthcare costs, and improve quality of life.

**Table 1:** Weight loss effect of the various pharmacological interventions.

Class of the drug	Example of the drug	Mechanism of action
Orlistat	Xenical	↓ cholesterol synthesis
Leptin analogue	Metreleptin	↓ appetite ↑ satiety
Lorcaserin	Belviq	↑ 5-HTC2, ↓ appetite
Rimonabant	Acomplia	↓ CB1 receptor, ↓ appetite
Phentermine/topiramate	Qsymia	↓ appetite
Naltrexone/Bupropion	Contrave	↓ Neurotransmitters reuptake, ↓ appetite
Setmelanotide	Imcivree	↑ MC4, ↓ appetite
Sibutramine	Meridia	↓ 5-HTC2 & norepinephrine uptake, ↓appetite
GLP-RA1, GIP/GLP-1 RA, GIP/GLP-1/Glucagon RA	Semaglutide, Liraglutide, Survodutide, Pemvidutide, Retatrutide, Orforglipron	↓ appetite ↑ satiety ↓ gastric motility

The up arrow (↑) means increased and the down arrow (↓) means decreased.

The need for a thorough comprehension of efficient interventions, the long-term effects of various treatment philosophies, and the ideal combination of tailored therapy are just a few of the knowledge gaps and conflicts that exist in obesity research. Currently, lifestyle interventions including physical activity and diet are the first-line management for those who are overweight or obese (14). Additionally, some patients might require behavioral therapy as an adjunct to lifestyle modification. However, their effectiveness is usually modest, and most patients will regain weight shortly after withdrawing from the intervention. For those who failed to achieve significant weight loss, pharmacotherapy can be used alone or as an add-on to lifestyle modifications as an effective combo but, the effect of bariatric surgery is surpassed their effectiveness (15). Thus, this review was aimed to provide a narrative overview of the current obesity treatment.

## Method

A systematic search for evidence in the literature was conducted. The search terms were initially developed and then searched combining the title and Medical Subject Headings (MeSH) for better evidence retrieval. The search was conducted using the following electronic databases: PubMed, EMBASE, MEDLINE, Scopus, Cochrane Library (CENTRAL) and Web of Science. Furthermore, we also searched for evidence of grey or unpublished evidence using the Open Grey Repository and Open Thesis Repository databases.

## Results

### Current therapeutic agents

#### Orlistat

Orlistat is a class of medication that inhibits gastric and pancreatic lipase and reduces the prandial absorption of fat by blocking triglyceride hydrolysis (16). By inhibiting pancreatic lipase, orlistat prevents the hydrolysis of dietary fats, leading to a reduction in the absorption of fat molecules. Consequently, undigested fats are excreted in the feces, resulting in decreased caloric intake and aiding in weight loss (16). Orlistat is a well-known drug used in obesity management with proven but low efficacy. A randomized open-label trial evaluated the orlistat effect on insulin resistance (IR) and compared with metformin and pioglitazone in obese women, treatment with orlistat has significantly reduced the IR compared to metformin and pioglitazone (17). Another study compared orlistat to metformin and lifestyle intervention in women with PCOS, treatment with orlistat showed significant improvement in lipid profiles and anthropometric measures (18). Furthermore, there was a significant reduction in androgen levels, parameters of insulin resistance (HOMA-IR) and IR (19, 20). In addition to its weight loss effect, orlistat can also modestly reduce blood pressure and plays a significant role in T2D prevention, this effect is possibly due to its weight reduction effect (21). The currently recommended orlistat dose is 120 mg up to 3 times a day and should be taken with food. However, even though its tolerability is high, it has significant side effects. The most common side effects associated with orlistat are pale stool, diarrhea, and flatulence (22). Even further, there is significant evidence that orlistat causes fat-soluble vitamin deficiencies (23). While orlistat might have desirable effects in the management of obesity, its effectiveness is relatively modest. Thus, it might be worth considering the other available options.

#### Liraglutide

**Figure 2:**
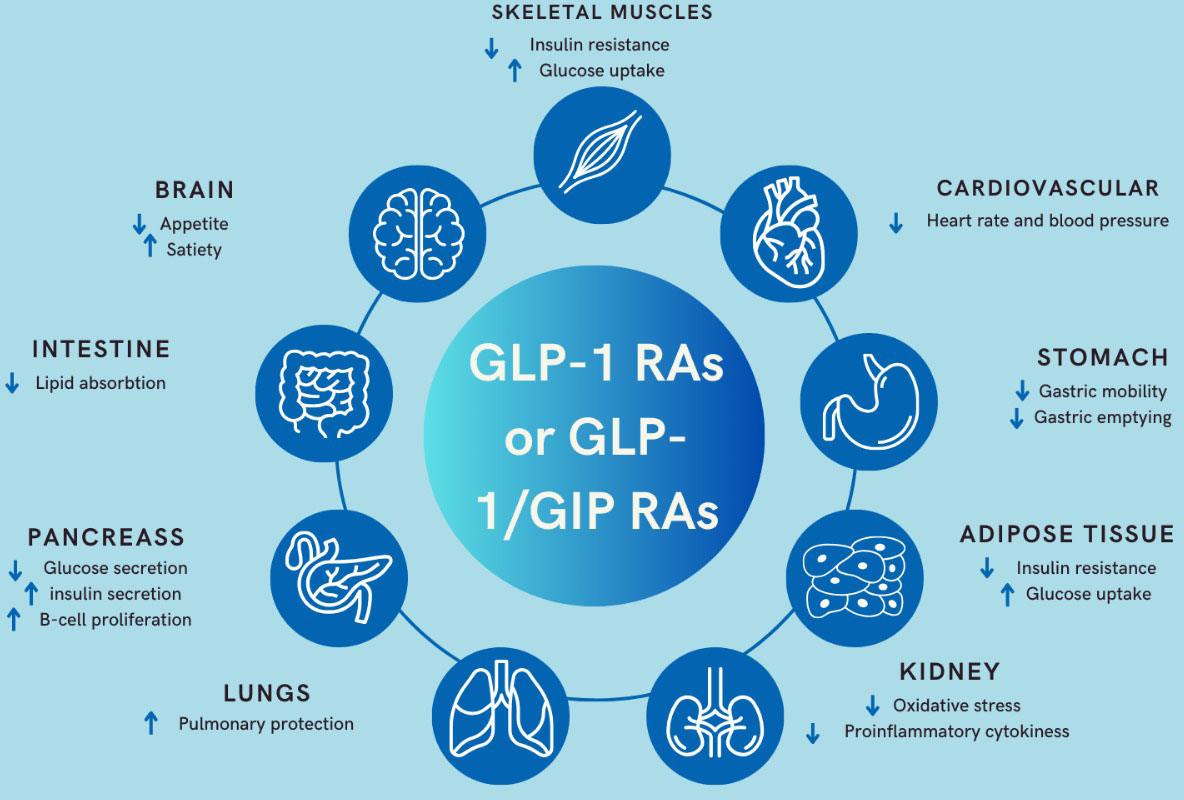
Mechanism of action of GLP-1 RAs and the dual GLP-1/GIP RAs .

Liraglutide is a medication used for the treatment of type 2 diabetes mellitus and obesity. Its mechanism of action involves mimicking the effects of a natural hormone called glucagon-like peptide-1 (GLP-1), which is released by the intestine in response to food intake. GLP-1 acts on GLP-1 receptors in various tissues, including the pancreas, liver, muscle, and brain. It enhances glucose-dependent insulin secretion and inhibits glucagon secretion through modulating pancreatic beta cells and alpha cells respectively. Additionally, it increases satiety and slows gastric emptying (**Figure 2**) (24). The SCALE trial involved the use of liraglutide, a GLP1 analogue, administered in a 3 mg subcutaneous weekly dose for 56 weeks. This trial encompassed a total of 3731 non-diabetic, obese patients who were randomly assigned to receive either a placebo (n=1244) or liraglutide (n=2487) in conjunction with lifestyle intervention. The trial’s results demonstrated that 63.2% of patients who received liraglutide experienced a weight loss of approximately 5% of their body weight, compared to 27.1% in the placebo group. Furthermore, 33.1% of patients on liraglutide achieved a weight loss of about 10% of their initial body weight, while only 10.6% of those on placebo achieved the same (24).

#### Trizepatide (dual GLP-1 & GIP RAs)

Tirzepatide is a novel dual GLP-1 receptor agonist (RA) and glucose-dependent insulinotropic polypeptide (GIP) with 39 amino acids, which acts on GLP-1 and GIP receptors. By simultaneously targeting both GIP and GLP-1 receptors, tirzepatide offers enhanced glycemic control and greater potential for weight loss compared to traditional GLP-1 receptor agonists alone (**Figure 2**). This dual mechanism of action makes tirzepatide an effective and promising treatment option for individuals with type 2 diabetes mellitus and obesity (25). Its half-life is approximately 5 days, allowing for once-a-week subcutaneous injection. In the SURPASS 1-5 trials, different dosages of Tirzepatide (5 mg, 10 mg, and 15 mg) demonstrated significant weight reduction in obese patients with type 2 diabetes mellitus (T2DM), especially when compared to placebo (SURPASS 1) (25), semaglutide 1 mg (SURPASS 2) (26), insulin degludec (SURPASS 3) (27), insulin glargine (SURPASS 4) (28), and placebo+ insulin glargine (SURPASS 5) (29). The overall weight loss ranged from 7.6 kg, 10.7 kg, to 12.9 kg with Tirzepatide 5 mg, 10 mg, and 15 mg, respectively.

The SURMOUNT 1-4 trials were designed to evaluate the effectiveness and safety of Tirzepatide as an adjunct to lifestyle interventions compared to a placebo in obese patients with or without T2DM. In all trials (SURMOUNT 1-4) (30, 31), treatment with Tirzepatide at various dosages was associated with significant body weight reduction, ranging between 12-20%, compared to placebo (3%) (30–32). SURMOUNT 5, which is expected to conclude in January 2025, is designed to compare Tirzepatide with Semaglutide 2.4 mg in overweight and obese adults with weight-related comorbidities. Tirzepatide is currently FDA-approved for obesity management.

#### Semaglutide

Glucagon-like peptide-1 (GLP-1) is an incretin hormone that stimulates insulin secretion from the pancreas and inhibits glucagon secretion in a glucose-dependent manner (33, 34). GLP-1 is primarily secreted by the L-cells in the small bowel (35). Its effects include reducing energy intake, suppressing hunger, and promoting satiety (36, 37). Studies involving obese and overweight patients, with or without diabetes, have shown that glucagon-like peptide-1 receptor agonists (GLP-1RAs) can reduce body weight and improve glucose control (24, 38).

Semaglutide is a GLP-1RA currently used in the treatment of obesity and diabetes. It is a GLP-1 analogue that has undergone three modifications to extend its half-life, allowing for once-weekly administration (39). The first modification involved substituting an amino acid at position 8 to reduce its susceptibility to degradation by the enzyme dipeptidyl peptidase-4 (DPP-4) (33). The second modification involved substituting the amino acid at position 34, resulting in derivatization at Lysine 26 (39).

Studies conducted as part of the Semaglutide Treatment Effect in People with Obesity (STEP) clinical trial development program evaluated the effects of a 2.4mg weekly dose of Semaglutide in overweight and obese individuals. Data from these STEP trials supported the approval of 2.4mg weekly Semaglutide for use in adults who are obese or overweight with weight-related comorbidities (40). This approval applies to Europe, the USA, Canada, and the UK (41–43). STEP trials 1, 3, 4, and 8 reported a mean weight loss of 14.9% to 17.4% from baseline to week 68 in obese and overweight patients without diabetes, associated with the use of weekly Semaglutide 2.4mg (44–47). Additionally, 69% to 79% of participants in these four trials achieved a weight loss of 10% or more (40). Furthermore, the STEP trials demonstrated improvements in waist circumference, lipid profiles, blood pressure, and C-reactive protein, all of which are cardiometabolic risk factors (44–48).

In the UK, the National Institute for Health, and Care Excellence (NICE) released guidelines for the use of Semaglutide in the management of obesity and overweight (49). These guidelines, issued in March 2023, recommend the use of Semaglutide for a maximum of 2 years in individuals with obesity/overweight who have one weight-related comorbidity and a body mass index (BMI) of at least 35 kg/m^2^. Patients with a BMI between 30 and 34.9 kg/m^2^ should meet the criteria for referral to a specialist weight management service, and it is expected that Semaglutide will be administered as part of a multidisciplinary team weight management program (49).

### Historical therapeutic agents

#### Phentermine/topiramate

In the year 2012, the Food Administration Authority (FDA) approved the combination of phentermine/topiramate as an adjunctive therapy to lifestyle modifications as a treatment for weight management in overweight and obese adult patients. It consists of two active ingredients: phentermine, a sympathomimetic amine, and topiramate, an antiepileptic drug. Phentermine acts as a sympathomimetic amine, primarily by increasing the release of norepinephrine in the brain which lead to appetite suppression and reduced food intake, contributing to weight loss. It also stimulates the release of dopamine and serotonin, neurotransmitters involved in regulating mood and appetite. Topiramate on the other hand, is thought to act on various neurotransmitter systems in the brain, including gamma-aminobutyric acid (GABA) and glutamate. By modulating these neurotransmitters, topiramate may reduce the rewarding properties of food, leading to decreased food intake and weight loss (50, 51). In a clinical study when the combination of phentermine/topiramate at the highest dose of 15/92 mg was used, it reduced the body weight by around 9.8-11% within 1 year compared to only around 7.5% of weight loss with its lower dose (7.5/46 mg) (50). However, in the effects of low-dose, controlled-release, phentermine/topiramate therapy on overweight and obese adults (CONQUER) trial, in which low dose (7.5 mg/46 mg) phentermine/topiramate was used in 2487 obese adults (994 in placebo arm vs 498 in phentermine/topiramate combination arm) for a total 56-weeks. Therefore, the results of the study showed significant weight reduction with the combination therapy compared with placebo (51). On the other hand, similar results were also seen with the phentermine/topiramate in severely obese adults (EQUIP) study, in which obese adults were randomized in three groups to receive either placebo (n=514), phentermine/topiramate 3.75 mg/23 mg (n=241) or phentermine/topiramate 15 mg/92 mg (n= 512) in conjunction with the standard lifestyle modifications as standard of care. After 56 weeks of treatment, the weight loss was achieved in 1.6% versus 5.1% and 10.9% in the placebo, phentermine/topiramate (3.75/23 mg) and phentermine/topiramate (15/92 mg), respectively (52). However, phentermine/topiramate combination therapy was associated with increased heart rate, mood changes, sleep disorders and gastrointestinal (53). Therefore, combination therapy has been denied marketing authorization by many countries due to its adverse effects.

#### Naltrexone/bupropion

Naltrexone is an opioid receptor antagonist which has a great affinity to bind to the µ opiate receptor, which influences eating behaviors. In observational studies, naltrexone alone has been shown to have the ability to antagonize dopamine secretion and reduce food intake and binge eating behavior. On the other hand, in human studies, naltrexone as a monotherapy has failed to show any consistent results. In the past few years, Naltrexone has been approved by the FDA as a treatment for alcohol and drug addiction (54). Conversely, Bupropion originally is an anti-depressant drug that was approved for the treatment of depression and is also often used in helping with smoking cessation. Bupropion acts by blocking the dopamine reuptake at the presynaptic cleft. Surprisingly, the main side effect of Bupropion was weight loss, hence its use as weight loss medication (55). Even though this agent was not principally approved for the management of obesity, several clinical trials suggested that the combination of these agents induces significant weight loss. Thus, the combination of Naltrexone/Bupropion has recently been approved for obesity treatment. In a recent double-blind placebo-controlled clinical trial CONTRAVE Obesity Research (COR-I and COR-II) in overweight and obese patients, a combination of N/B demonstrated more or less similar weight loss effect (-8.1% and -8.2%, respectively) compared to placebo (56, 57). Moreover, in the COR-Behavioral Modification (COR-BMOD) trial, where patients were treated with the combination of N/B in adjunct with intensive behavioral modification program or placebo, treatment with the N/B+BMOD showed significant weight loss compared to placebo + BMOD (-11.5% vs -7.3%; *p <*0.01, respectively) (58).

#### Setmelanotide

Setmelanotide is an anorexigenic medication and acts as a melanocortin-4 receptor (MC4R) agonist, it was approved by the FDA in the year 2020 for the management of genetic obesity. The drug showed it is the ability to help restore appetite control, however; it did not correct the hereditary problems that caused obesity (53). Therefore, setmelanotide is indicated in patients with genetic obesity due to either the defect in the pro-opiomelanocortin (POMC) gene, Leptin receptors (LEPR)gene, and the proprotein convertase subtilisin/kexin type 1 (PCSK1) gene. In principle, appetite is usually controlled by the satiety center in the hypothalamus which is itself regulated by the regulatory hormone such as leptin under several regulatory pathways. Therefore, setmelanotide was shown to restore the defect in these pathways and thus, reduce appetite and induce energy expenditure (59).

#### Metreleptin

Leptin is a hormone primarily produced by the adipose tissues, it plays a role in regulating energy balance by affecting appetite, inducing satiety, and managing behavioral feeding. When fat stores are adequate, leptin levels rise, signaling to the brain to reduce appetite, increase energy expenditure, and maintain metabolic homeostasis. Metreleptin is marketed as a leptin analogue, it was approved by the FDA in the year 2014 as a substitute to deficient leptin in patients with lipodystrophy. In individuals with generalized lipodystrophy, leptin levels are typically very low or absent due to the lack of adipose tissue. This deficiency disrupts normal energy balance regulation, leading to severe metabolic abnormalities such as hyperphagia (excessive hunger), insulin resistance, hypertriglyceridemia, and hepatic steatosis (53). Its route of administration is subcutaneously daily (53). In a recent non-randomized crossover study of 25 patients with lipodystrophy who were leptin-deficient, metreleptin was associated with increasing the resting metabolic rate and, improved the metabolic parameters (60). Moreover, metreleptin showed beneficial effects in improving insulin resistance, liver steatosis and hypogonadism (61). However, recently metreleptin treatment has been associated with the development of leptin antibodies which has a nullified effect on its action.

#### Sibutramine

Sibutramine is an appetite suppressant and is often used as an adjunct alongside lifestyle intervention in obesity management. Its mechanism of action is to block the reuptake of neurotransmitters such as serotonin, dopamine and norepinephrine (62). This inhibition leads to a reduction in appetite and subsequently, a reduction in food consumption (63). Despite its effectiveness, it has been reported that sibutramine might be associated with significant cardiovascular risk and potential strokes. These potential adverse events have led Sibutramine to withdraw from the market due to safety concerns (64).

#### Rimonabant

Rimonabant is a selective blocker of the cannabinoid receptor 1 (CB1) and is used as a treatment for obesity management which reduces appetite (65). The mechanism of rimonabant involves blocking the activity of cannabinoid receptors, specifically CB1 receptors, which are abundant in the central nervous system and peripheral tissues. CB1 receptors are part of the endocannabinoid system, which plays a crucial role in regulating appetite, energy balance, and metabolism. Activation of CB1 receptors by endocannabinoids, such as anandamide and 2-arachidonoylglycerol, increases appetite, promotes food intake, and enhances the storage of energy as fat. By acting as a CB1 antagonist/inverse agonist, rimonabant blocks the binding of endocannabinoids to CB1 receptors, thereby inhibiting their activity. Overall, the mechanism of rimonabant involves modulating the endocannabinoid system to suppress appetite, promote weight loss, and potentially improve metabolic health (65). A recent trial found that rimonabant could significantly reduce alanine aminotransferase (ALT) and enhance weight loss in obese women with polycystic ovary syndrome (PCOS) who did not have non-alcoholic fatty liver disease (NAFLD) (66). Another study in which rimonabant was compared to metformin for treating obese women with PCOS, found a significant increase in the glucose-dependent insulinotropic polypeptide (GIP) with rimonabant compared to metformin (67). However, it is important to note that rimonabant has been associated with severe psychiatric problems, such as depressive disorders, mood changes, and suicidal ideation (68). Due to these side effects, rimonabant has been withdrawn from the markets for obesity management.

#### Lorcaserin

Lorcaserin is a weight loss medication that was approved by the FDA for obesity management in patients with BMI > 30 kg/m^2^ or in those with BMI < 27 kg/m^2^ and diabetes, dyslipidaemia, or hypertension. Its mechanism of action is to activate the serotonin receptor (5-HT_2c_) in the hypothalamus which will subsequently suppress appetite and thus associated with significant weight loss ranging from 3-5% particularly when used alone or added to lifestyle interventions (69, 70). However, due to serious concerns about its possible association with cancer, it was withdrawn from the market in 2020 (71).

### On the horizon therapeutic agents

There are a few promising therapeutic agents currently under investigation some of them act centrally by reducing appetite and enhancing satiety while others act peripherally. Those agents work within the central nervous system to reduce appetite and enhance feelings of fullness, which can help control calorie intake. They may also affect other metabolic processes. Some of these agents fall into the category of GLP-1 receptor agonists, which have shown promise in improving metabolic parameters. The effects of these agents on weight, lipid profiles, blood pressure, and glucose metabolism can contribute to overall metabolic health. These agents can also target gastric motility or the movement of food through the digestive system. By slowing down gastric motility, they can prolong the feeling of fullness and reduce the rate at which calories are absorbed. This can lead to reduced calorie intake and potentially contribute to weight loss.

An example of these medications is Survodutide, a selective synthetic dual agonist of the glucagon receptor (GCGR) and the glucagon-like peptide-1 (GLP-1) receptor. As a dual agonist, it may provide benefits by targeting multiple pathways involved in glucose metabolism and appetite regulation. Survodutide is currently in a phase III clinical trial for non-alcoholic steatohepatitis (NASH), a liver condition often associated with metabolic issues like obesity and type 2 diabetes. This agent’s potential to address NASH is of significant clinical interest (72). Retatrutide is described as a triple agonist, acting on the glucose-dependent insulinotropic polypeptide (GIP), glucagon-like peptide-1 (GLP-1), and glucagon receptors. Phase II clinical trials for retatrutide are underway, and it’s mentioned to have substantial weight reduction effects. GIP and GLP-1 are both hormones involved in regulating insulin and appetite, and the addition of glucagon receptor activation may provide a comprehensive approach to metabolic health (73). Orforglipron is an oral non-peptide GLP-1 receptor agonist designed for weight reduction. The fact that it’s orally administered can be an advantage over injectable GLP-1 RAs like liraglutide and semaglutide. Phase II clinical trials are ongoing, and promising weight reduction effects are noted (74). Recently, Pemvidutide has been a dual agonist of GLP-1 and glucagon. This agent is being investigated in phase II clinical trials for the treatment of NASH, and obesity with and without type 2 diabetes. The dual mechanism of action may offer unique benefits for weight loss and metabolic improvement. These agents represent exciting developments in the field of metabolic and obesity-related therapies. The ability to simultaneously address multiple aspects of metabolic health, including weight reduction, lipid profile improvement, and glucose metabolism, is a promising approach in the management of conditions such as obesity, type 2 diabetes, and NASH.

### Surgical interventions

#### Bariatric surgery

The fundamental basis for bariatric surgery is to achieve weight loss in patients who have not been able to lose weight through non-surgical means (75). Specific criteria established by consensus indicate that bariatric surgery is appropriate for patients with a BMI > 40 kg/m^2^ and for patients with a BMI > 35 kg/m^2^ who have associated comorbidities (76). The most common bariatric surgery procedure is Roux-en-Y Gastric Bypass (RYGB), in which the stomach is transected to create a gastric pouch of approximately one-ounce capacity (77). Sleeve gastrectomy (SG) involves resecting around 80% of the stomach to create a tubular stomach. Additionally, biliopancreatic diversion with duodenal switch, along with implantable devices, are a few other examples of bariatric procedures (75). Over the past few years, several studies have shown superior weight loss effects for bariatric surgery. A systematic review and meta-analysis that evaluated the percentage of weight loss (EWL%) and diabetes remission demonstrated superior effects one year after surgery compared to the standard of care. Surprisingly, these effects were still evident three years after the surgery. Moreover, there is significant evidence supporting the use of bariatric surgery to achieve diabetes remission in patients with T2DM (78). However, there are complications associated with bariatric surgery, including surgery-related issues and nutritional deficiencies (79).

## Limitations of the review

Findings from studies comparing GLP-1 agonists and bariatric surgery may not be generalizable to all populations, as they often involve specific patient groups or settings. Furthermore, the long-term effects of GLP-1 agonists and bariatric surgery on weight loss and other outcomes are not yet fully understood, and more research is needed to assess their sustainability. On the other hand, both GLP-1 agonists and bariatric surgery can have adverse effects, such as gastrointestinal symptoms with GLP-1 agonists and surgical complications with bariatric surgery. These risks should be carefully considered when offering either option to patients. Bariatric surgery can be costly and may not be accessible to all patients, while GLP-1 agonists may also be expensive and require ongoing treatment, which can impact their feasibility for some individuals. Although combining GLP-1 agonists and bariatric surgery shows promise, there are challenges in determining the optimal timing, dosing, and patient selection for this approach.

### Future directions

Developing personalized treatment approaches based on genetic factors and other patient characteristics could enhance the effectiveness of GLP-1 agonists and other treatments for obesity and diabetes. Continued research into the development of new GLP-1 agonists with improved efficacy and fewer side effects holds promise for better patient outcomes. Further exploration of combination therapies involving GLP-1 agonists, bariatric surgery, and other treatments could lead to synergistic effects and improved weight loss outcomes. Conducting long-term studies to evaluate the sustained effects of GLP-1 agonists and bariatric surgery on weight loss, glycemic control, and other outcomes is crucial for understanding their long-term benefits and risks. Addressing healthcare policy and access issues to ensure that effective treatments for obesity and diabetes, including GLP-1 agonists and bariatric surgery, are accessible to all patients who could benefit from them, is also important.

## Conclusion

Treatments based on incretin hormone GLP-1 agonists are rapidly evolving. While weight loss was initially discovered as a secondary outcome of using GLP-1 agonists in the treatment of T2DM, it has since been compared to surgical intervention, specifically bariatric surgery, as a means of weight reduction. Based on studies, both interventions have demonstrated improvements in glycemic control, lipid profiles, and weight loss. However, bariatric surgery generally yields superior outcomes compared to GLP-1 agonists. Nevertheless, it’s essential to note that bariatric surgery is an invasive procedure only suitable for a specific group of patients. Therefore, a promising approach involves combining both interventions to achieve enhanced weight loss results, particularly in patients who have gained weight post-surgery. Looking forward, developing new agonists that target specific genes based on genetics may hold the key to further advancements in this field.

## Author contributions

AM: Writing – original draft. KH: Writing – original draft. HB: Writing – original draft. JA-F: Software, Visualization, Writing – original draft. AA: Writing – original draft. RA-H: Writing – original draft. MA: Writing – review & editing. FA-M: Writing – review & editing. MA-F: Conceptualization, Writing – review & editing. JA: Conceptualization, Writing – review & editing.
